# Encouragement of Vaccination in Prussia

**Published:** 1836-04

**Authors:** 


### ENCOURAGEMENT OF VACCINATION IN PRUSSIA.

By an official notification from the department of the Prussian government having charge of ecclesiastical, educational, and medical affairs, issued at Berlin on the 14th March, 1835, it is intimated to the different governments of the kingdom, that a silver medal has been struck at the royal Mint, for the purpose of being presented to the parents and relations of children who shall bring them forward, when vaccinated, with the view of supplying the public establishments with vaccine matter; and the public vaccination boards are permitted to use their funds for the purchase of the medals to be so employed.

*Rust's Magazine*, B. 45, Heft 1, 1835.

